# Seasonal variation in net ecosystem CO_2_ exchange of a Brazilian seasonally dry tropical forest

**DOI:** 10.1038/s41598-020-66415-w

**Published:** 2020-06-11

**Authors:** Keila R. Mendes, Suany Campos, Lindenberg L. da Silva, Pedro R. Mutti, Rosaria R. Ferreira, Salomão S. Medeiros, Aldrin M. Perez-Marin, Thiago V. Marques, Tarsila M. Ramos, Mariana M. de Lima Vieira, Cristiano P. Oliveira, Weber A. Gonçalves, Gabriel B. Costa, Antonio C. D. Antonino, Rômulo S. C. Menezes, Bergson G. Bezerra, Cláudio M. Santos e Silva

**Affiliations:** 1 Climate Sciences Post-graduate Program, Federal University of Rio Grande do Norte, Av. Senador Salgado Filho, 3000, Zip Code 59078-970 Lagoa Nova, Natal Brazil; 2 Meteorology Post-graduate Program, Federal University of Campina Grande, Rua Aprígio Veloso, 882, Zip Code 58429-900, Universitário, Campina Grande, Brazil; 3 National Institute of Semi-Arid, Av. Francisco Lopes de Almeida, s/n, Zip Code 58434-700, Serrotão, Campina Grande, Brazil; 4 Department of Atmospheric and Climate Sciences, Federal University of Rio Grande do Norte, Av. Senador Salgado Filho, 3000, Zip Code 59078-970 Lagoa Nova, Natal Brazil; 5 Institute of Biodiversity and Forests, Federal University of Western Pará, UFOPA, Santarém, Pará Brazil; 6 Federal University of Pernambuco, Department of Nuclear Energy, Recife, Pernambuco Brazil

**Keywords:** Biogeochemistry, Climate sciences

## Abstract

Forest ecosystems sequester large amounts of atmospheric CO_2_, and the contribution from seasonally dry tropical forests is not negligible. Thus, the objective of this study was to quantify and evaluate the seasonal and annual patterns of CO_2_ exchanges in the *Caatinga* biome, as well as to evaluate the ecosystem condition as carbon sink or source during years. In addition, we analyzed the climatic factors that control the seasonal variability of gross primary production (GPP), ecosystem respiration (R_eco_) and net ecosystem CO_2_ exchange (NEE). Results showed that the dynamics of the components of the CO_2_ fluxes varied depending on the magnitude and distribution of rainfall and, as a consequence, on the variability of the vegetation state. Annual cumulative NEE was significantly higher (p < 0.01) in 2014 (−169.0 g C m^−2^) when compared to 2015 (−145.0 g C m^−2^) and annual NEP/GPP ratio was 0.41 in 2014 and 0.43 in 2015. Global radiation, air and soil temperature were the main factors associated with the diurnal variability of carbon fluxes. Even during the dry season, the NEE was at equilibrium and the *Caatinga* acted as an atmospheric carbon sink during the years 2014 and 2015.

## Introduction

CO_2_ concentration has a high interannual variability due to its absorption by terrestrial ecosystems (carbon sinks)^1–5^. However, despite this variability, data show a systematic increase in CO_2_ throughout the years^6,7^. In South America, the Amazon forest is an example of a terrestrial carbon sink (considering its 20-year mean behavior), although it has occasionally behaved as CO_2_-neutral or even a carbon source in the last years^8^.

Interannual variability and trends in CO_2_ sinks are controlled by different biogeographic regions. The annual mean behavior of sinks is controlled mainly by highly productive lands, such as wet tropical forests (i.e. the Amazon forest)^5^. On the other hand, semiarid environments control the global scale trends observed in the last few decades^9,10^. Despite its prominent role, there is still much to be studied and investigated regarding CO_2_ exchanges in these regions, which are still much less understood than wet forests or croplands^5,10^. According to the literature^10^, gaps in understanding CO_2_ exchanges in these environments have limited our ability to understand and predict interannual and decadal variations on global scale carbon cycle. There are a few inherent difficulties when quantifying CO_2_ exchanges in semiarid environments, such as the rapid expansion of some of its areas due to climate change and anthropic activities^11,12^. Studies show that some regions in South America are becoming more arid, such as the Amazon^13,14^; the Brazilian semiarid region, dominated by the *Caatinga* biome, which is a seasonally dry tropical forest (SDTF)^15–17^ and the *Cerrado*, which is a Brazilian savanna-type vegetation^18^.

Interannual variability of CO_2_ absorption by terrestrial sinks is mainly associated with land use changes and meteorological factors, because carbon balance is strongly related to their high spatial and temporal variability. Among them, rainfall plays an important role due to its remarkable seasonality in semiarid regions^19–21^. In these ecosystems, the availability of natural resources such as water, plant biomass, litter and soil nutrients is modulated by the occurrence of rainfall^22^. During periods of rainfall abundance, there are more nutrients available in the soil, resulting in a faster, more efficient absorption by plants, increasing leaf development and productivity^23,24^. In periods of rainfall scarcity, reductions in enhanced vegetation index (EVI) values in seasonally dry tropical ecosystems reflect the reduction in leaf area due to leaf loss during the dry season. Thus, leaves undergo senescence and CO_2_ absorption reduces to a minimum^21,22^. The crucial role of rainfall in the variability of terrestrial carbon sinks was put in evidence by anomalies in observed data from the year 2011^19,25,26^. The most plausible cause for this anomaly was the expansion of semiarid vegetation in the southern hemisphere, particularly in the Australian savannas, associated with a global rainfall anomaly between the years 2010 and 2011 caused by the persistency of a strong La Niña event^19^. During 2010 and 2011, the carbon sink in Australia was of 0.97 Pg^26^. In the following seasons, this sink reduced with rainfall, reaching 0.08 Pg in 2012 and 2013, when rainfall was below average^26^. This case also shows the important influence that semiarid ecosystems might have on global carbon exchange dynamics. The interannual variability in global scale terrestrial sinks is also associated with tropical nighttime warming^20^, due to the intensification of ecosystem respiration. This seeming sensitivity of respiration to temperature variations^27^, particularly nighttime temperature, suggests that carbon stocked in tropical forests might be vulnerable to a warmer future scenario.

This is an alarming remark, especially regarding the *Caatinga* biome, which occupies an area of over 800,000 km², possesses a vast endemic biodiversity and is acknowledged as one of the most important wildlife areas of the planet^21,28,29^. It is the main ecosystem in the Brazilian semiarid region, where projections indicate an increase of up to 1 °C in mean air temperature during the next three decades (2020–2050) as well as a decrease of up to 20% in rainfall amount^21,30^. Recent observations point towards a systematic increase in climate extreme events indices associated with nighttime temperature in the Brazilian semiarid^31,32^. Furthermore, there is evidence of an intensification of aridity and the expansion of semiarid lands in the Northeast Brazil^33,34^, which may directly influence on the dynamics of the *Caatinga* tropical forest.

Similarly to what is observed in most semiarid ecosystems around the globe, studies on the dynamics of CO_2_ exchange in the *Caatinga* biome are still scarce. Thus, there is an urgent need to quantify biosphere-atmosphere carbon exchange in the *Caatinga* in order to better understand its role in the regional climate system. One of the most important steps in this process is the investigation of the impacts of environmental factors in carbon exchange. Our hypothesis is that the *Caatinga* can function as a strong sink for carbon if compared to other dry forests, presenting high carbon use efficiency as a result of low ecosystem respiration even in the dry season. Therefore, the present study aims to quantify and evaluate the seasonal and annual patterns of CO_2_ exchange and the annual carbon balance in the *Caatinga* biome, as well as its condition as carbon sink or source. Furthermore, we aim to analyze the climatic factors that control the seasonal variability of CO_2_ flux components (gross primary production – GPP, ecosystem respiration – R_eco_ and net ecosystem exchange – NEE) and to determine the patterns of NEE seasonal variability in respect to CO_2_ flux components (GPP e R_eco_). Measurements were carried out through an eddy covariance system during the years 2014 and 2015.

## Results

### Meteorological conditions

**Figure 1:**
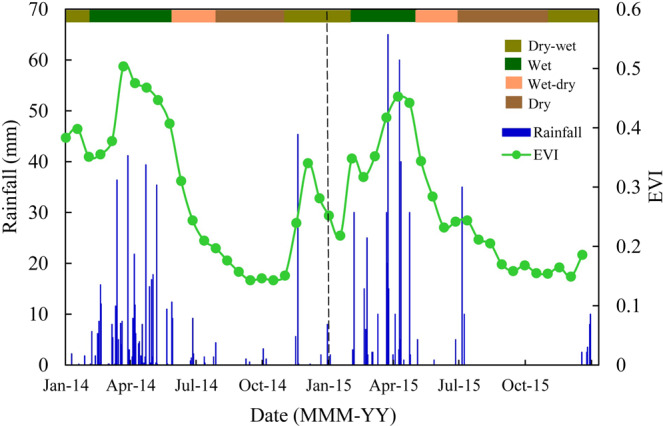
Rainfall and EVI in the Caatinga (ESEC-Seridó) during the years of 2014 and 2015.

Annual accumulated rainfall in 2014 and 2015 was of 513 mm and 466 mm, respectively, while the annual climatological value is of 758 mm (Table 2). This characterizes the study period as of below-average rainfall. The series of daily accumulated rainfall (Fig. 1) highlights the remarkable seasonal variation in rainfall, although the studied years presented some particularities. In 2014, highest rainfall amounts were observed during the months from March to May and the highest daily value was of 42 mm. In 2015, there were fewer but more intense rainy days, with values larger than 60 mm in April. Similarly, daily rainfall amounts larger than 35 mm were observed in June 2015, which did not occur in 2014. Another important pattern is the absence of rainfall from August to November 2015. Overall, rainfall was better distributed in 2014, but in 2015 more daily extreme events were registered.

**Table 1:** Species, family, life-form, common name, relative frequency (RtF, %) and importance value (IV) sampled in the study area by Santana *et al*. (2016). All scientific names of species were obtained in The Plant List platform*.

Species	Family	Common name	Life-form	RtF	VI
*Caesalpinia pyramidalis* Tul.	Leguminosae	Catingueira	Tree	10.60	54.30
*Aspidosperma pyrifolium* Mart.	Apocynaceae	Pereiro	Tree	11.40	51.20
*Croton blanchetianus* Baill.	Euphorbiaceae	Marmeleiro	Shrub	9.76	50.10
*Anadenanthera colubrina* (Vell.) Brenan	Leguminosae	Angico-vermelho	Tree	10.20	20.30
*Mimosa tenuiflora* (Willd.) Poir.	Leguminosae	Jurema-preta	Shrub	6,91	16,96
*Combretum leprosum* Mart.	Combretaceae	Mufumbo	Shrub	8,13	15,83
*Piptadenia stipulacea* (Benth.) Ducke	Leguminosae	Jurema-branca	Shrub	6.10	14.40
*Commiphora leptophloeos* (Mart.) J.B.Gillett	Burseraceae	Umburana	Tree	6.10	13.70
*Jatropha mollissima* (Saraiva *et al*.) Baill.	Euphorbiaceae	Pinhão bravo	Shrub	2.60	13.40
*Erythroxylum pungens* O. E. Schulz	Erythroxylaceae	Rompe-gibão	Shrub	6,50	12,20
*Caesalpinia ferrea* C. Mart.	Leguminosae	Jucá	Tree	2.03	8.150
*Croton heliotrophiilious* Kunth	Euphorbiaceae	Velame	Herbaceous	6.77	3.66
*Handroanthus impetiginosus* (Mart. ex DC.) Mattos	Bignoniaceae	Ipê roxo	Tree	5.37	3.66
*Cnidoscolus quercifolius* Pohl	Euphorbiaceae	Faveleira	Tree	3.71	2.03
*Bignonia corymbosa* (Vent.) L.G.Lohmann	Bignoniaceae	Bugi	Liana	3.17	2.03
*Bauhinia cheilantha* (Bong.) Steud.	Leguminosae	Mororó	Shrub	2.97	1.22
*Amburana cearensis* (Allemao) A.C.Sm.	Leguminosae	Cumaru	Tree	0.81	1.90
*Senna macranthera var. micans* (Nees) H.S.Irwin & Barneby	Leguminosae	Canafístula	Shrub	0.81	1.35
*Chamaecrista hispidula* (Vahl) H.S.Irwin & Barneby	Leguminosae	Maria-preta	Shrub	0.81	1.01
*Cereus jamacaru* DC.	Cactaceae	Mandacaru	Tree	0.41	0.62
*Cynophalla flexuosa* (L.) J.Presl	Capparaceae	Feijão-bravo	Shrub	0.41	0.49
*Lantana camara* L.	Verbenaceae	Chumbinho	Herbaceous	0.41	0.46

^*^Available on http://www.theplantlist.org/. Accessed in Oct 12, 2018.

**Table 2:** Weather variables observed in the ESEC-Seridó during experimental period. Total annual rainfall (P, mm), mean annual temperature (T_a_, °C), annual incoming solar radiation (R_g_, MJ m^−2^) and annual mean vapor pressure deficit (VPD, kPa).

Year	P	T_a_	T_s_	R_g_	VPD
2014	513	28.9	31.4	8030	1.7
2015	466	29.5	33.9	8249	1.9
Climatology	758	26.8	—	—	1.3

In the drier year (2015), T_a_ was higher (29.5 °C) if compared to the mean 2014 value (28.9 °C), although both were higher than the mean climatological value (26.8 °C). Soil temperature was higher (33.9 °C) in 2015 if compared to 2014 (31.4 °C) (Table 2). In 2014, annual integrated R_g_ was of 8,030 MJ m^−2^, which is slightly inferior to the 2015 value of 8,249 MJ m^−2^. This, in turn, agrees with the previous analysis regarding temporal rainfall distribution in 2014. The mean annual value for the VPD was of 1.7 kPa in 2014 and 1.9 kPa in 2015. In 2015, there were a higher number of days in which VPD was larger than 2.5 kPa. It is worth mentioning that T_a_, R_g_ and VPD mean annual values were all higher than the climatological values in the study area (Table 2), which characterizes the period as warmer, with more incident radiation, and drier than the average state.

The response of the *Caatinga* to rainfall can be analyzed through the EVI plot (Fig. 1). The variability of the index and rainfall behave accordingly. Based on these results we defined the seasons as: wet, wet-dry, dry and dry-wet, which were considered in the CO_2_ fluxes analysis. During the wet season, EVI reached its peak values, around 0.42 (2014) and 0.38 (2015). On the other hand, EVI consistently decreases during the wet-dry transition season until reaching its lowest values (0.16) during the dry season.

### Seasonal and annual variability of CO_2_ fluxes

**Figure 2:**
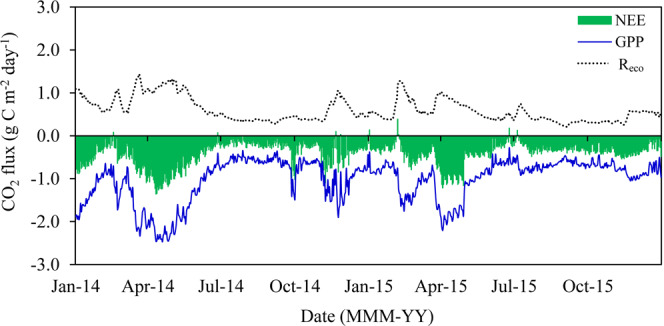
Daily cumulative net ecosystem CO_2_ exchange (NEE), gross primary production (GPP) and ecosystem respiration (R_eco_) during the study period in the Caatinga (ESEC-Seridó). Carbon uptake was denoted as negative and carbon release was denoted as positive.

Daily cumulative GPP, R_eco_ and NEE time series show the existence of a clear seasonal variability (Fig. 2). Table 3 shows seasonal and annual accumulated means of each flux variable.

**Table 3:** Mean ± standard deviation (g C m^−2^ y^−1^) and accumulated (g C m^−2^) seasonal and annual net ecosystem exchange (NEE), gross primary production (GPP), ecosystem respiration (R_eco_) and ecosystem carbon-use efficiency (NEP/GPP ratio) fin the study period.

Year	Variable	Statistics	Dry-wet transition	Wet season	Wet-dry transition	Dry season	Annual
2014	NEE	Mean	**−0.50** ± **(0.05)**	−0.70 ± (0.33)	−0.25 ± (0.04)	**−0.26** ± **(0.03)**	**−0.46** ± **(0.03)**
		Sum	**−45.9**	−84.0	−14.9	**−24.2**	**−169.0**
	GPP	Mean	**−1.20** ± **(0.08)**	**−1.68** ± **(0.10)**	−0.73 ± (0.06)	**−0.64** ± **(0.04)**	**−1.14** ± **(0.06)**
		Sum	**−110.6**	**−200.8**	−44.3	**−59.0**	**−414.7**
	R_eco_	Mean	**0.70** ± **(0.04)**	**0.98** ± **(0.04)**	0.48 ± (0.03)	**0.39** ± **(0.01)**	**0.68** ± **(0.03)**
		Sum	**64.7**	**117.0**	29.4	**34.7**	**246.0**
	NEP/GPP	Ratio	**0.41**	0.42	0.34	**0.41**	0.41
2015	NEE	Mean	**−0.28** ± **(0.03)**	−0.63 ± (0.07)	−0.30 ± (0.03)	**−0.36** ± **(0.01)**	**−0.40** ± **(0.02)**
		Sum	**−17.9**	−56.0	−27.1	**−44.0**	**−145.0**
	GPP	Mean	**−0.79** ± **(0.03)**	**−1.43** ± **(0.08)**	0.78 ± (0.04)	**−0.71** ± **(0.02)**	**−0.92** ± **(0.04)**
		Sum	**−49.0**	**−127.0**	72.0	**−86.0**	**−334.0**
	R_eco_	Mean	**0.51** ± **(0.02)**	**0.80** ± **(0.04)**	0.49 ± (0.02)	**0.34** ± **(0.02)**	**0.52** ± **(0.02)**
		Sum	**30.9**	**71.3**	44.8	**42.0**	**189.0**
	NEP/GPP	Ratio	**0.36**	0.44	0.38	**0.51**	0.43

Bold values are significantly different between each season in 2014 and its correspondent season in 2015 at the 0.05 level.

The GPP and NEE increased at the onset of the wet season and reaching peak values in April 2014 and 2015 (Fig. 2), which GPP declined until reaching values lower than −1.0 g C g C m^−2^ d^−1^ in the dry season. Mean seasonal GPP in 2014 varied from −0.64 g C m^−2^ d^−1^ (dry season) to 1.68 g C m^−2^ d^−1^ in the wet season (Table 3). In 2015, GPP values presented a similar trend, ranging from −0.71 g C m^−2^ d^−1^ (dry season) to −1.43 g C m^−2^ d^−1^ in the wet season (Table 3).

The onset of the wet season also resulted in periods with larger R_eco_ fluxes, with peaks higher than 1 g C m^−2^ d^−1^ (Fig. 2), gradually declining with the reduction in precipitation. Ecosystem respiration was significantly larger (p < 0.01) in 2014 than in 2015, both in the dry season and the wet season (Table 3).

In order to identify seasonal differences in the period of maximum and minimum NEE, we calculated the mean value between 10:00–12:00 (midday) and 22:00–00:00 (nighttime) (Table 4). Mean seasonal estimates of midday NEE in 2014 ranged from −5.1 µmol m^−2^ s^−1^ in the dry season to −12.3 µmol m^−2^ s^−1^ in the wet season, with an annual mean of −8.6 µmol m^−2^ s^−1^. In 2015, values ranged from −6.2 µmol m^−2^ s^−1^ in the wet-dry season to −11.6 µmol m^−2^ s^−1^ in the wet season, with an annual mean of −7.6 µmol m^−2^ s^−1^ (Table 4). Mean seasonal estimates of nighttime NEE presented a similar pattern. Annual accumulated NEE was significantly higher in 2014 (−169.0 g C m^−2^) if compared to 2015 (−145.0 g C m^−2^) (p < 0.05; Table 3). Furthermore, carbon-use efficiency, defined as the NEP/GPP ratio varied from 0.34 to 0.51 between seasons. The relationship between annual NEE and GPP was of 0.41 in 2014 and 0.43 in 2015 (Table 3).

**Table 4:** Annual and seasonal mean ± standard deviation of midday (10:00–12:00) and nighttime (22:00–00:00) net ecosystem CO_2_ exchange (NEE; µmol m^−2^ s^−1^) measured in the *Caatinga*, Brazilian Semiarid, during 2014 and 2015.

Year	NEE	Dry-wet	Wet	Wet-dry	Dry	Annual
2014	Midday	−9.3 ± 3.7	−12.3 ± 4.2	−5.6 ± 2.2	−5.1 ± 1.8	−8.6 ± 4.5
	Nightime	2.5 ± 1.1	2.9 ± 0.9	1.7 ± 0.4	1.4 ± 0.3	2.1 ± 1.0
2015	Midday	−6.3 ± 1.0	−11.6 ± 3.6	−6.2 ± 1.4	−6.3 ± 1.0	−7.6 ± 3.1
	Nightime	1.8 ± 0.5	2.7 ± 0.9	1.6 ± 0.4	1.4 ± 0.3	1.8 ± 0.8

### Correlation patterns between CO_2_ fluxes components and climatic factors

**Figure 3:**
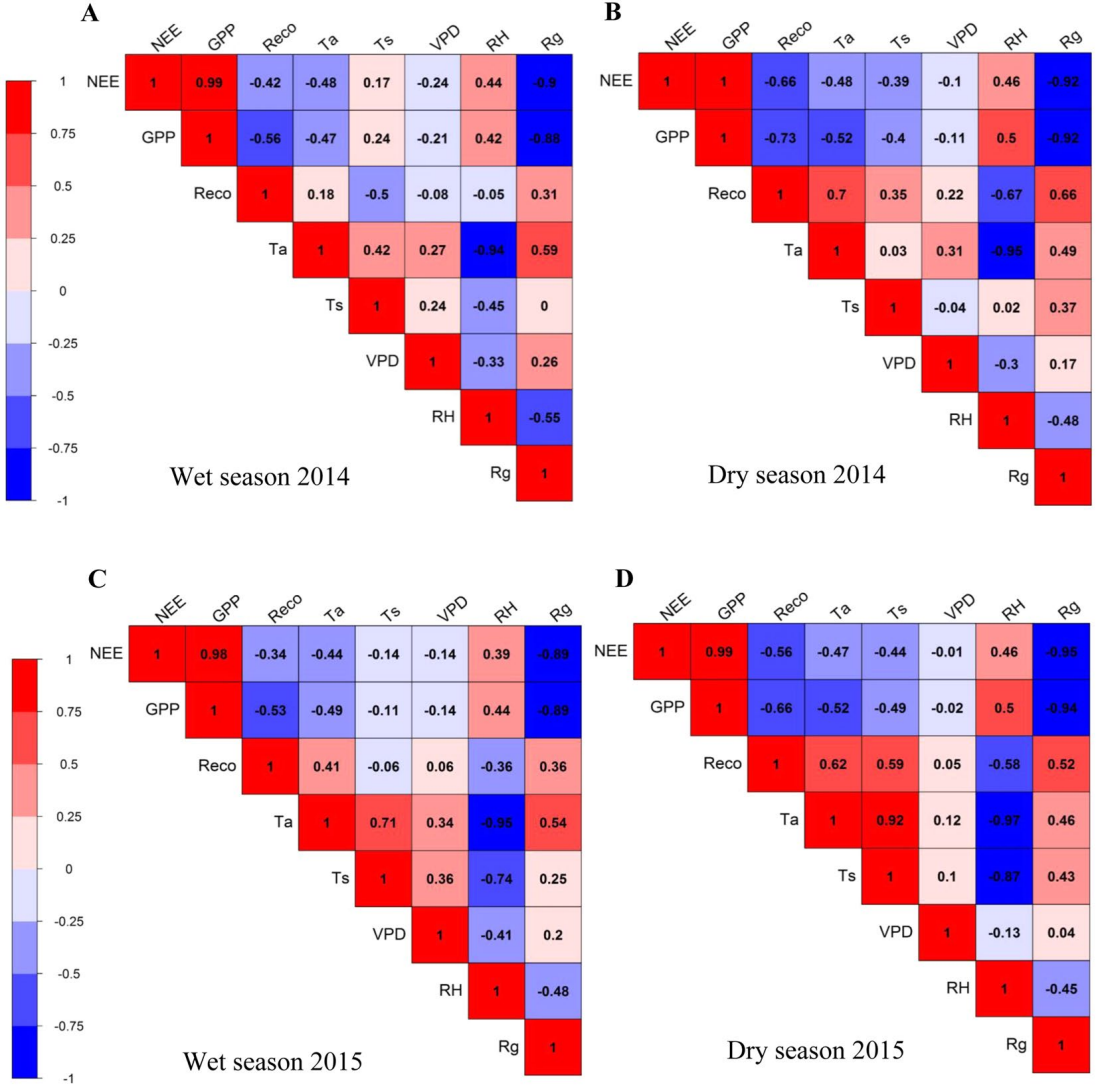
Heat map based on correlation matrix between net ecosystem CO_2_ exchange (NEE) gross primary production (GPP), ecosystem respiration (R_eco_), air temperature (T_a_), soil temperature (T_s_), vapor pressure deficit (VPD), relative humidity (RH), global radiation (Rg) for the wet season (**A**,**B**, respectively) and dry season (**C**,**D**, respectively) of 2014 and 2015 in the Caatinga (ESEC-Seridó). Warm colors represent positive correlation and cool colors represent negative correlation based on Pearson’s correlation test (p < 0.05).

The correlation matrix heatmap (Pearson’s correlation test) between the wet and dry season data from 2014 to 2015 show relevant patterns between NEE, GPP, R_eco_ and the meteorological variables observed at the surface (Fig. 3). With no exceptions, NEE is highly correlated with GPP (p < 0.01) in all study period. Ecosystem respiration is negatively correlated with NEE and GPP (p < 0.01), indicating that the ecosystem increases carbon assimilation and respiration simultaneously. However, correlations between GPP and R_eco_ are higher than those between NEE and R_eco_. It is interesting to note that GPP has a stronger negative correlation with R_eco_ in the dry season (R = −0.73 in 2014 and R = −0.66 in 2015) than in the wet season (R = −0.56 in 2014 and R = −0.53 in 2015).

**Figure 4:**
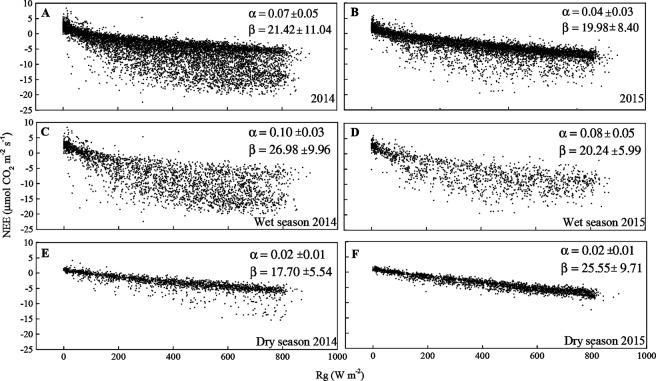
Net ecosystem CO_2_ exchange (NEE) in relation to global radiation (R_g_) for all seasons of the years 2014 (**A**) and 2015 (**B**), during the wet season (**C**,**D**, respectively) and dry season (**E**,**F**, respectively) of 2014 and 2015 in the Caatinga (ESEC-Seridó).

In all seasons, NEE and GPP are negatively correlated with global radiation (Rg) (p < 0.01, Fig. 3). Light response curves relating daytime CO_2_ fluxes and solar radiation show that, for similar light levels, more CO_2_ was absorbed in 2014 than in 2015 (Fig. 4). The response between wet and dry seasons is different, regarding both slope and shape of the curvature. In the dry season, the dynamic changed, and NEE magnitude was smaller, which can be observed by regarding the difference in the slope of the curve (Fig. 4).

Positive correlations are observed between NEE, GPP and RH (p < 0.01), with larger correlation coefficients observed in the dry season of both study years. Ecosystem respiration is negatively correlated with RH (p < 0.05), suggesting that an increase in air relative humidity causes a decrease in ecosystem respiration. However, correlations between VPD and NEE, GPP or R_eco_ are not significant (p > 0.05, Fig. 3).

Significant correlations are observed between NEE, GPP, R_eco_, T_a_ and T_s_. Net ecosystem exchange and GPP are negatively correlated with T_a_ (p < 0.01, Fig. 3) in both the wet season and the dry season. However, NEE and GPP responses to T_s_ could only be significantly perceived in the dry season (p < 0.05). Ecosystem respiration is more strongly correlated with T_a_ and T_s_ in the dry season (Fig. 3).

**Figure 5:**
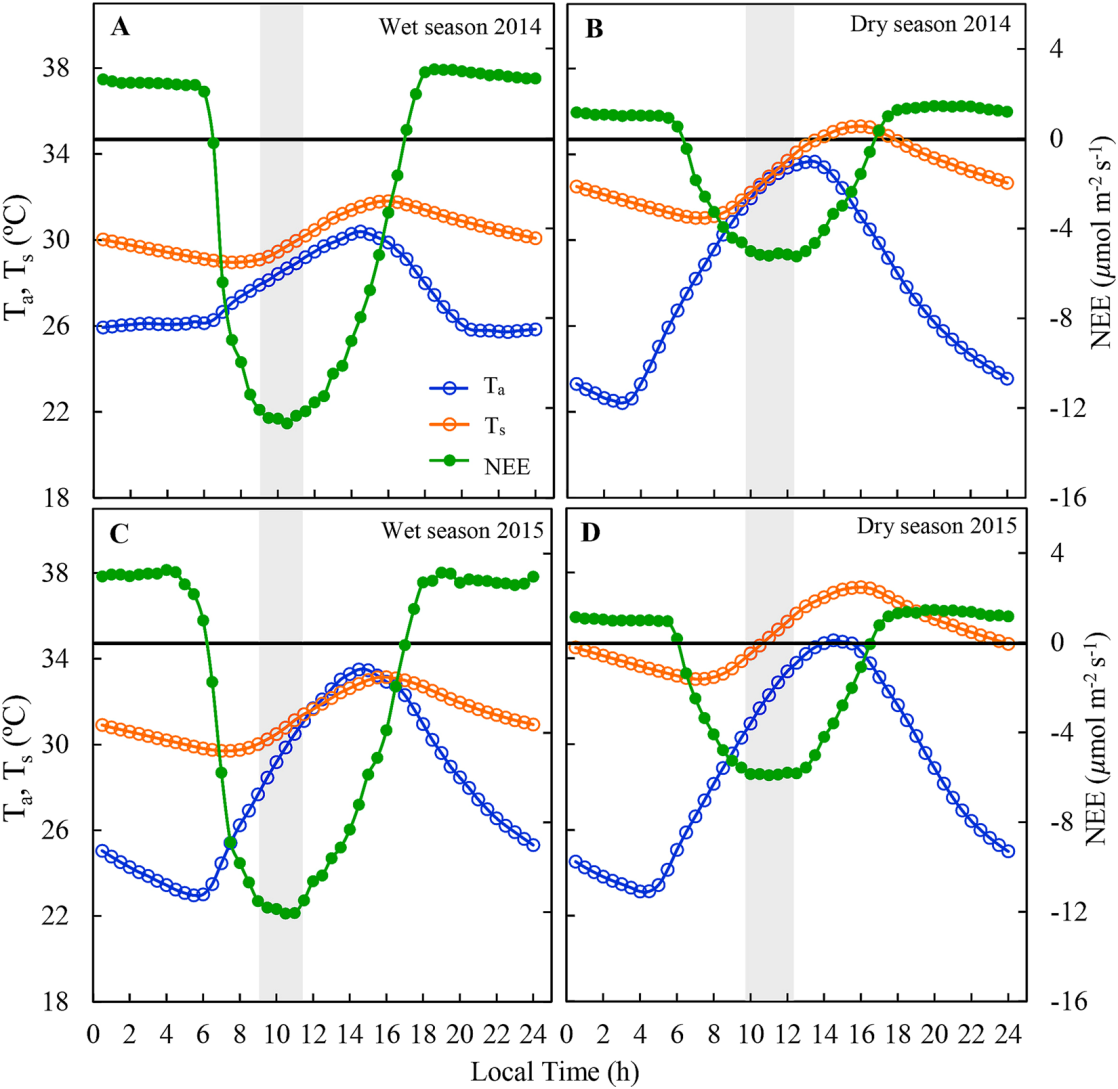
Diurnal variations in net ecosystem CO_2_ exchange (NEE), air temperature (T_a_) and soil temperature (T_s_) during the wet season (**A**,**B**, respectively) and dry season (**C**,**D**, respectively) of 2014 and 2015 in the Caatinga (ESEC-Seridó). The gray box represents the time of the day in which NEE presented its peak.

**Figure 6:**
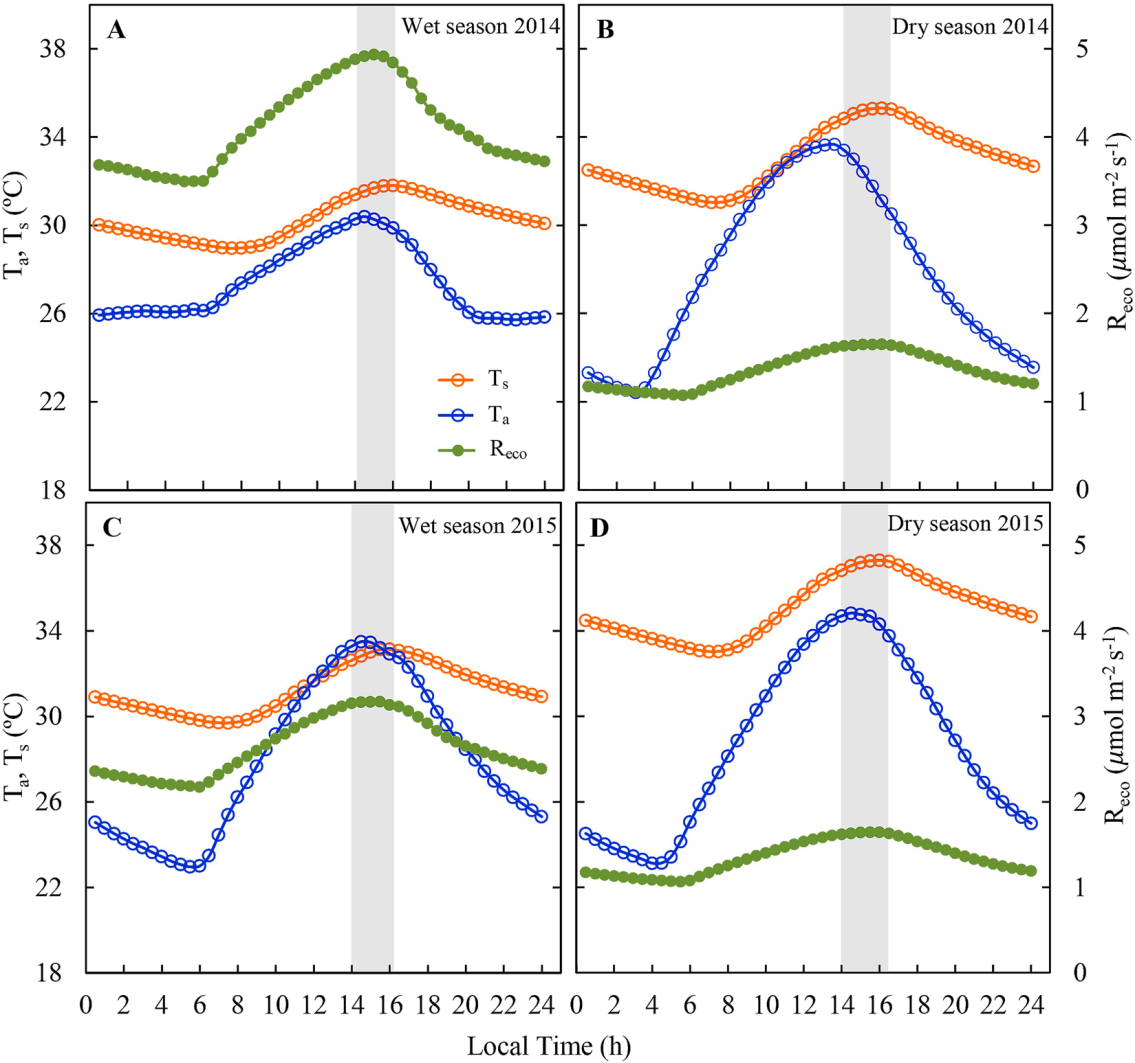
Diurnal variations in ecosystem respiration (R_eco_), air temperature (T_a_) and soil temperature (T_s_) during the wet season (**A**,**B**, respectively) and dry season (**C**,**D**, respectively) of 2014 and 2015 in the Caatinga (ESEC-Seridó). The gray box represents the time of the day in which R_eco_ presented its peak.

The responses of NEE and R_eco_ diurnal cycles to air and soil temperature are presented in Fis. 5 and 6. Following previous studies^12^ that observed a lag between peak GPP and peak monthly mean air temperature in four different regions of the globe. Based on that, we elaborated curves in order to identify the existence of this lag at the daily scale, regarding both air and soil temperatures (Figs. 5 and 6). Net ecosystem exchange diurnal peaks in the wet season occurred between 28 and 30 °C, and in the dry season between 30 and 33 °C (Fig. 5), gradually declining with the increase in T_a_ and T_s_ after noon. The largest R_eco_ values were observed between 14:00 and 16:00 h, which coincides with maximum air (32 to 35 °C, Fig. 6) and soil (31 to 37 °C, Fig. 6) temperature peaks.

**Figure 7:**
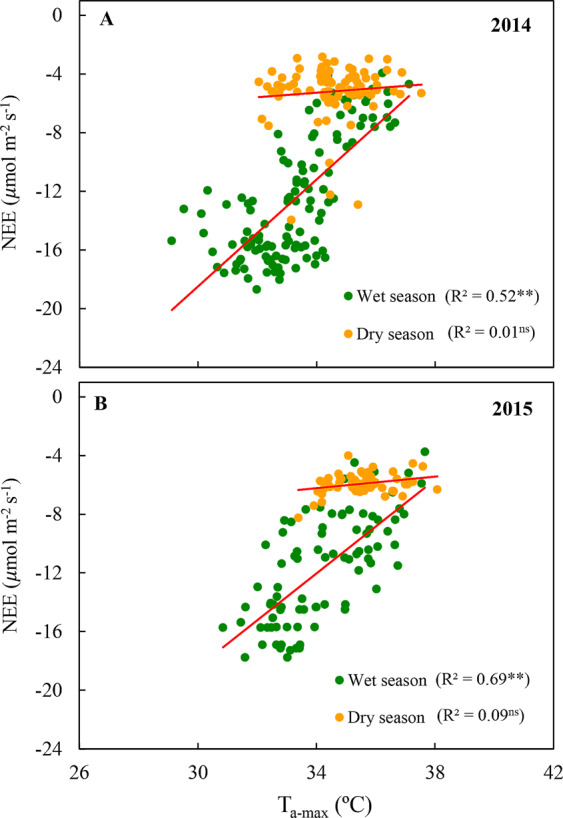
Relationship between daily averages of midday (10:00–12:00) net ecosystem CO_2_ exchange (NEE) and maximum air temperature (T_a-max_) during the wet season and dry season (2014, **A** and 2015, **B**) in the Caatinga (ESEC-Seridó).

In order to determine the effects of maximum temperatures (T_a-max_) on net ecosystem CO_2_ exchange, we analyzed midday NEE in respect to maximum daily air temperature (Fig. 7). During the wet season of 2014 and 2015, NEE decreased linearly with the increase in T_a-max_, which ranged from 31 to 38 °C (Fig. 7). On the other hand, there is no direct correlation between NEE and T_a-max_ in the dry season of the study years (p > 0.05).

## Discussion

The meteorological variables observed during the experimental period show extremely dry conditions over the Northeast Brazil. This condition can be explained mainly by the coupling of a warm El Niño Southern Oscillation phase, particularly in 2015, and positive anomalies in North Atlantic sea surface temperature. These factors contributed to the northern displacement of the Intertropical Convergence Zone, which caused a reduction in annual rainfall over the Northeast Brazil and also part of the Amazon during the 2012–2016 period^35^. This ended up effecting other meteorological variables such as incoming solar radiation, wind speed, minimum and maximum temperatures, soil temperature and air relative humidity^21^.

We also observed that CO_2_ fluxes (GPP, R_eco_ and NEE) were strongly influenced by the effects of seasonal climatic factors (Fig. 2). Gross primary production and NEE sharply rose (negative values, controlled by CO_2_ assimilation) after leaf expansion (higher EVI) due to the onset of the wet season, when soil moisture is recharged. Thus, during the wet season, GPP rates exceeded R_eco_ and the *Caatinga* acted as a carbon sink. The higher vegetation cover in the biome during 2014 (Fig. 1) also incurred in an increase of CO2 assimilation and thus NEE was more negative that year, in which rainfall was better distributed.

On the other hand, the reduction in GPP and NEE values (close to zero) with the decline of precipitation is closely related to the reduction in the EVI. This represents leaf senescence in the *Caatinga*, a mechanism of drought resilience which limits photosynthetical activity to the few semi-deciduous plant species which succeed in keeping their leaves the entire year. During the dry season, trees gradually suffer from the decline in soil water content which leads to stomatal closure and the reduction of stomatal conductance and leaf transpiration, which in turns limit CO_2_ assimilation and further reduces net photosynthesis. These modulations of the physiological mechanisms of the *Caatinga* plants have been previously reported^36–38^, and therefore we can infer that photosynthetical characteristics such as stomatal conductance, kinetics of the Rubisco enzyme and electron-transfer, can influence on the seasonal variability of CO_2_ fluxes.

Similar to the variation observed in NEE during the experimental period, R_eco_ also followed a seasonal pattern (Fig. 2). Ecosystem respiration promptly responded to rainfall. Peak values during the onset of the wet season might be associated not only with the physiological processes inherent to the development of new leaves but also with soil physical conditions in the *Caatinga*. Organic matter decomposition and microbial activity increase in the wet season, which in turn increases R_eco_ since it consists of both autotrophic and heterotrophic respiration^39–41^.

The low respiration and GPP of the *Caatinga* is evidenced by analyzing data in Table 3. During 2014 and 2015, annual R_eco_ values were of 246 and 189 g C m^−2^, respectively, while annual GPP values were 414.7 and 334.0 g C m^−2^, respectively, which in turn implies a R_eco_/GPP ratio of approximately 0.55. This value is considerably lower than values reported in the literature^42,43^. The low respiration observed in semiarid regions is partially associated with reduced organic carbon stocks in relation to humid regions^44^. Observed organic carbon stock in the *Caatinga* biome is almost 25% lower than the values found in the Amazon; 65% lower than the values found in the *Cerrado* biome (Brazilian savanna); and almost 80% lower than the values observed in the Atlantic Forest^45^. Besides the low organic carbon stock, low plant respiration also plays a role, mainly during periods of water scarcity. Under these conditions, plants maintain respiration at basal levels, i.e., low rates of autotrophic respiration^36–38^, therefore reducing the overall respiration of the ecosystem. Another factor that probably contributed to the reduced respiration rates observed in the *Caatinga* refers to the fact that the study was carried out during drought years. Thus, the water scarcity conditions observed in this period limited the biological activity of plants and soil microorganisms, which in turn limit respiration.

Compared to other types of dry forests (Table 5), the *Caatinga* can be considered a major carbon sink with an average assimilation of −1.57 t C ha^−1^ y^−1^ (−157 g C m^−2^ y^−1^) in the study period (Table 5) and remarkable interannual variability. For instance, a smaller net carbon gain was reported in semiarid savannas in California^46,47^. On the other hand, large CO_2_ fluxes were observed in the semiarid savanna of western Africa, which was attributed to a high fraction of C4 species and alleviated water stress conditions that resulted in compensatory effects in vegetation growth^41^. According to the literature^48^ also observed high ecosystem productivity rates in the Mulga forest, due to significant increases in rainfall (565 mm y^−1^) and soil water storage during a La Niña event that influenced the climate in the entirety of Australia in 2011. However, in subsequent years the Mulga forest behaved as a minor carbon source (25 g C m^−2^ y^−1^) with the reduction of rainfall (193 mm y^−1^) and then as a minor carbon sink (12 g C m^−2^ y^−1^) with the increase in rainfall (295 mm y^−1^) after the second hydrological year that followed^25^.

**Table 5:** Overview of reported net ecosystem CO_2_ exchange (NEE) in various tropical savanna and forest ecosystems worldwide.

Vegetation type, site	Period	NEE (g C m^−2^ y^−1^)	GPP (g C m^−2^ y^−1^)	R_eco_ (g C m^−2^ y^−1^)	Mean Rainfall	Mean Annual Temperature (°C)	Source
STDF Caatinga, northern of Brazilian Semiarid	January – December, 2014 January – December, 2015	−169–145	414.7 334.0	246 189	513 mm y^−1^ 466 mm y^−1^	28.9 29.5	In this study In this study
STDF Caatinga, north central of Brazilian Semiarid	March, 2014 – March, 2015	−282	—	—	430 mm y^−1^	23–32	Silva *et al*. (2017)
Campo sujo savanna in central Brazil	June, 1998 – July, 1999	−288	1,272	984	1,017 mm y^−1^	22.5	Santos *et al*. (2003)
Campo sujo savanna in central Brazil	March, 2011 – December, 2013	−242 – −357	—	—	1,160 mm y^−1^	26.3	Zanella De Arruda *et al*. (2016)
Oak/grass Savanna, California (USA)	April, 2001– October, 2006	−98.0	1,070	972	562 mm y^−1^	16.5	Ma *et al*. (2007)
Low tree and shrub Savana, Senegal, west Africa	August, 2010 – December, 2013	−271	1,076	773	524 mm y^−1^	28.3	Tagesson *et al*. (2015)
Nazinga: Shrub and tree mix, Kayoro: Tall grasses, West Africa	October, 2012 – January, 2013	Nazinga: −387 Kayoro: 108	1,725.1 781.3	1,337.8 889.3	320–1,100 mm y^−1^	29.0	Quansah *et al*.(2015)
Arid-zone Acacia savanna woodland, Mulga, Central Australia	August, 2010 – July, 2011	−259	—	—	565 mm y^−1^	21–34	Eamus *et al*. (2013)
Arid-zone Acacia savanna woodland, Mulga, Central Australia	August, 2012 – August, 2013 September, 2013 – August, 2014	25–12	—	—	193 mm y^−1^ 295 mm y^−1^	21–34 21–34	Cleverly *et al*. (2016)
Tropical rainfall, East-central Amazonia, Brazil Tropical rainfall, Central Amazonia, Brazil Neotropical rainforests, French Guiana Tropical peat swamp forest in Kalimantan, Indonesia	2002–2011 2000–2004 2004–2014 2002–2003	−57–171–157 375	3,425 3,234 3,720 3,209	3,391 3,033 3,560 3,584	7.5 mm d^−1^ 4.5 mm d^−1^ 8.0 mm d^−1^ 6.0 mm d^−1^	25.0 25.0 26.0 27.0	Fu *et al*. (2018) Fu *et al*. (2018) Fu *et al*. (2018) Fu *et al*. (2018)

Negative values indicate net ecosystem carbon uptake, while positive values indicate net ecosystem carbon loss.

Differences between the results found in the studies^49^ – net carbon loss between 242–357 g C m^−2^ y^−1^ – and^50^ – net carbon gain of −288 g C m^−2^ y^−1^ – in a savanna in central Brazil were attributed to distinct soil textures associated with a lower soil water storage capacity between the studied sites. This relationship between CO_2_ fluxes and soil texture was also observed in the literature^51^ in different savannas in Sudan. In this case, the Nanzinga site, which gained carbon (−387 g C m^−2^ y^−1^), presented a soil with higher water storage capacity when compared to the Kayoro site, which lost carbon (108 g C m^−2^ y^−1^). A summary of the net CO_2_ exchange in different tropical rainforests was presented in the study^52^, which revealed that ecosystems with larger carbon assimilation by photosynthesis (GPP > 3,000 g C m^−2^ y^−1^) usually have a smaller net carbon uptake or even carbon losses because the intensity of ecosystem respiration is similar to GPP (Table 5). Curiously, annual NEE observed in the *Caatinga* in the present study was higher than that of east-central Amazon, and comparable to that of central Amazon and Neotropical rainforests, as shown in Table 5.

The NEP/GPP (where NEP is the net ecosystem production, i.e., NEP = -NEE, as described in the studies^43,53^) ratio values, which are direct measures of carbon use efficiency at the ecosystem level, were 0.41 in 2014 and 0.43 in 2015 (Table 3). These values are higher than the usual range of 0.05 to 0.35 found in different forest types under different climate conditions around the globe^12,42,43,53^. However, high carbon use efficiency in semiarid forests has been previously reported and is attributed to low carbon loss through respiration^12^. Various studies have reported that a higher NEP/GPP ratio implies higher carbon transfer from the atmosphere to terrestrial biomass. Indirectly, this ratio represents how much carbon is emitted to the atmosphere through vegetation autotrophic respiration^53,54^. In the present study, the *Caatinga* proved to be carbon-use efficient, even when subjected to water stress. For comparative purposes, NEP/GPP ratio values found in our study are relatively larger than the ones found in the literature^55^ in the Amazon forest (0.34), in 12 European pine forest sites (0.17), in the entire global FLUXNET network (0.17), and in the Yatir semiarid forest (Israel) (0.27)^12,42^.

Net ecosystem exchange proved to be highly sensitive to small shifts in the components of CO_2_ fluxes (GPP and R_eco_) (Fig. 3). Several studies have reported that a small shift in any of these components may be crucial in determining whether it will behave as a carbon source or sink^39,56,57^. Correlations found in the present study between CO_2_ flux components suggest that the seasonal variability of NEE is also controlled by the behavior of GPP and R_eco_. Robust correlations between GPP and NEE in all seasons imply that productivity and net carbon uptake increase simultaneously in the *Caatinga*. The inverse relationship between NEE, GPP and R_eco_ indicates that gross primary productivity and net carbon sequestration of the ecosystem increase with ecosystem respiration, especially in the dry season, possibly due to seasonal contrast in nitrogen deposition in the plant litter and leaf senescence, as discussed in the literature^58^.

Control of the energy balance components in NEE is expected because NEE is R_g_-dependent. The NEE showed a typical hyperbolic relationship with R_g_ in the wet season, while in the dry season the relationship was linear, saturating at about 800 W m^−2^ (Fig. 4). The response curves of NEE to R_g_ indicate that the maximum photosynthetic activity was lower under dry conditions when compared to the wet season. This suggests that drier conditions induced changes in photosynthetic processes. Furthermore, one can notice that there are more random variations during the wet season, which might be associated with the use of an open path sensor, as suggested in the literature^59^. These results are consistent with previous studies on different ecosystems that identified strong R_g_ control in the seasonal and interannual variability of GPP and NEE^58,50^. Data from the present study show the relationship between air and soil temperature and the seasonality of NEE, GPP and R_eco_ in the Caatinga. In the study period, the occurrence of higher values of T_a_ during 2015 was attributed to the occurrence of a very strong El Niño event^21^. The direct influence of air temperature on the seasonal variability of CO_2_ fluxes is possibly related to increased water stress due to the intensification of the drought period in the Northeast Brazil^31^. With this, results raised an important question: in addition to the seasonal changes in rainfall and in the structure and functioning of the *Caatinga*, do variations in air and soil temperature have an impact on the carbon balance of this biome?

The *Caatinga* underwent a change in its NEE and R_eco_ responses to variations in air and soil temperature during the 2014 and 2015 wet and dry seasons, showing high respiration response during the wet period compared to low respiration rates in the dry season (Fig. 6). In addition, R_eco_ was more sensitive to air temperature during the dry season with higher carbon losses between 32 and 34 °C. However, there is no remarkable increase in R_eco_ with the increase in maximum air temperatures under water deficit conditions. For many biomes, it has been demonstrated that temperature has a strong influence on R_eco_ and is a relevant factor controlling the metabolism of plants and decomposers^50,60^.

When NEE was analyzed in relation to T_a_, it can be seen that it increased rapidly until 10:00 h and decreased after midday, in anticipation of the period of higher air temperature (13:00 to 14:00 h; Fig. 5). Carbon assimilation in the wet season reached its peak in the temperature range between 28 and 30 °C, whereas in the dry season the highest values of NEE were registered in the temperature range between 30 and 34 °C and decreased thereafter. While NEE declined with an increase in T_a_ and T_s_ above a moderate range for each season, VPD had no significant effect on the components of the CO_2_ flux. In addition, CO_2_ absorption was more sensitive to variations in T_a_ during the day, but T_s_ was the main driver of net CO_2_ changes in the ecosystem at night. The decline in CO_2_ absorption with the increase in the optimal T_a_ range during dry conditions can be explained by the increase in stomatal resistance, which reduces CO_2_ exchange between the stomates and the atmosphere^61^. In addition, the lag between the peaks of NEE and T_a_ can represent a mechanism of the *Caatinga* plants to prevent leaf-level warming, which causes a decrease in the efficiency of Rubisco carboxylation by promoting photorespiration, thus reducing photosynthesis^62^.

The decrease in NEE values during the dry season (Fig. 5) is offset by the lower R_eco_ rates (Fig. 6), which boosted the *Caatinga* to be a carbon sink under extreme climate conditions. This suggests that R_eco_ is the main driver of net CO_2_ exchanges in the *Caatinga*, especially in the dry season. Our results are consistent with other studies. For example, other studies^57^ showed that total ecosystem respiration, and not GPP, controls the interannual variation of the carbon balance in boreal forests. As reported in the literature^63^ nighttime R_eco_ increases with rainfall, which may result in lower net carbon uptake in perennial pastures during years of above-average rainfall. In a *Caatinga* site, has been reported^64^ that lower CO_2_ fluxes in the dry season and net CO_2_ emissions were significantly influenced by soil temperature, showing an inverse relationship.

Recent studies show that warmer tropical nighttime temperatures are associated with lower net absorption of terrestrial carbon^20^. Thus, trends of increase in maximum temperature above the threshold of 34 °C^31^, especially in the wet season (Fig. 7), can probably have a negative impact on the NEE, since the increase in air temperature can cause a decline in productivity, as well as an anticipation and reduction of diurnal peaks of CO_2_ absorption in the *Caatinga*.

## Summary and conclusions

The seasonal and annual patterns of CO_2_ exchange and the annual carbon balance were analyzed during 2014 and 2015, on a preserved fragment of the *Caatinga* biome, which is a seasonally dry tropical forest in the Brazilian semiarid region. In general, results showed that the carbon balance dynamics of the *Caatinga* biome is intrinsically related to rainfall seasonality. Carbon sequestration is maximum in the wet months and minimum during the dry months due to the scarcity of water in the soil. Despite the impact of rainfall on improving productivity in the *Caatinga*, the ecosystem also releases carbon to the atmosphere, although carbon losses were not meaningful. Even during the dry season, NEE was in equilibrium and the *Caatinga* functioned as an atmospheric CO_2_ sink during 2014 and 2015. The sensitivity of carbon exchanges to rainfall seasonal variability demonstrates the coupling between carbon and hydrological fluxes in the *Caatinga*, indicating that seasonal changes in weather conditions are important. These results show that the length of the wet period and total accumulated rainfall modulate the behavior of carbon fixation rates in the biome.

The results presented in this study have a crucial to elucidate (and demystify) uncertainties about the actual role of the *Caatinga* biome in the regional and global carbon balance. We found that the estimates of ecosystem respiration (CO_2_ lost to the atmosphere) are reasonably low. On the other hand, carbon-use efficiency is high. Thus, the balance (fixation of CO_2_) is superior and/or comparable to some tropical rainforests, such as the Amazon, and this extremely important result needs to be taken into consideration in public policy projects that deal with the preservation of native areas of *Caatinga*. We also point out that the two studied years were of extreme drought, and therefore the wet seasons were shorter than usual, especially in 2015. Thus, it is expected that during years with more intense and better distributed rainfall the *Caatinga* will be even more efficient in using/assimilating carbon and accumulating biomass.

The correlation analysis indicated that the seasonal variability of NEE is strongly influenced by productivity (GPP) both in the wet season and in the dry season. Nevertheless, the lower rates of R_eco_ in the dry season seem to be the driver of net CO_2_ exchange in the *Caatinga*. In addition, air and soil temperatures were the main factors responsible for the diurnal variability of carbon fluxes. The reduction of NEE after noon was explained by the physiological responses of *Caatinga* plants to the increase in maximum temperature, especially during the wet season. These results point out that changes in maximum air temperature are likely to affect not only actual carbon balance in the *Caatinga* biome but also the modeling of ecosystem carbon balances. Therefore, changes in the dynamics of dry forests should be considered in coupled climate models. In addition, future studies should investigate the acclimatization of *Caatinga* trees to increases in air temperature.

## Material and methods

### Site description

The study was carried out during the years of 2014 and 2015 in a fragment of *Caatinga* biome in the Seridó Ecological Station (ESEC-Seridó) (6°34’42”S, 37°15’05”W, 205 m above sea level) located between the cities of Serra Negra do Norte and Caicó, in the Rio Grande do Norte state, Brazilian semiarid region. The ESEC-Seridó is a conservation unit of the *Caatinga* biome, managed by the Chico Mendes Institute for Biodiversity Conservation (ICMBio), with an area of 1,163 ha of preserved *Caatinga*, characterized by a dry, xerophyte forest with sparsely distributed shrubs and small trees (less than 7 meters in height), and herb patches which thrive only during the wet season and are reduced to plant litter during the dry season^21^.

Regarding the relative frequency (RtF) and the importance value (IV) of the species that occur in the study area^65^, the *Leguminosae* and *Euphorbiaceae* families present the highest count of individuals (Table 1). There is a balance between the number of arboreous and shrub species, although arboreous species are predominant (RtF higher than 50%). Shrub species have a RtF of around 23%. Three of the four dominant species are arboreous (*Caesalpinia pyramidalis* Tul., *Aspidosperma pyrifolium* Mart., and Anadenanthera colubrina (Vell.) Brenan) and one is shrub (*Croton blanchetianus* Baill.). The three arboreous species have a combined RtF larger than 40%. Most of the species listed in Table 1 are deciduous and semi-deciduous.

Predominant soil type is Lithic Neosol with sandy loam and sandy clay loam textures, shallow, rocky, and with low fertility mainly due to low levels of organic matter and low water retention capacity^66^. Soil characteristics for the 0–20 cm layer in the study site are: soil organic carbon 10.65 g kg^−1^, density 1.41 kg dm^−3^, and pH 5.9^66^. Average concentration of total soil P is 196 mg kg^−1^ and biological atmospheric N_2_ fixation is estimated to vary between 3 and 11 kg N ha^−1^ y^−^1 in mature *Caatinga*^45^. The region’s climate is low latitude and altitude semiarid (BSh) according to the Köppen classification^67^. Wet season occurs between January and May with a mean annual rainfall below 700 mm, mean air temperature of 25 °C and relative air humidity around 60% (30-yr mean)^21,68^. Terrain slope varies from 1 to 3 degrees.

### Instrumentation and measurements

The ensemble of instruments used consists of an eddy covariance (EC) system installed in a tower with 11 m of height, managed by the Brazilian National Institute of Semiarid (INSA) and part of the National Observatory of Water and Carbon Dynamics in the Caatinga Biome (NOWCDCB) network. Measurements were conducted from 01 January 2014 to 31 December 2015, retrieving high frequency (10 Hz) and low frequency (5 s) data.

High frequency data consist of CO_2_ and water vapour concentration measurements and the three wind speed components (${u}_{y}$, ${u}_{z}$, ux), retrieved using an Integrated CO_2_/H_2_O Open-Path Gas Analyzer & 3D Sonic Anemometer (IRGASON, Campbell Scientific, Inc., Logan, UT, USA). Atmospheric pressure was measured by an Enhanced Barometer PTB110 (Vaisala Corporation, Helsink, Finland). Air temperature was measured by a HMP155A probe (Vaisala Corporation, Helsink, Finland). All high frequency data were sampled at a 10 Hz frequency and stored in a memory stick coupled to a datalogger model CR3000 (Campbell Scientific, Inc., Logan, UT, USA).

Low frequency data consist of net radiation (R_n_), soil heat flux (G), soil temperature (T_s_), air temperature (T_a_) and relative humidity (RH), besides rainfall. Rn measurements were carried out through a net radiometer model CNR4 (Kipp & Zonen B. V., Delft, The Netherlands). Soil heat flux was measured by two heat plates model HFP01SC (Hukseflux Thermal Sensors, Delft, The Netherlands), installed at a 0.05 m depth. T_a_ and RH data were measured using a temperature and relative humidity probe model HMP45C (Vaisala Corporation, Helsink, Finland). T_s_ was measured using a 108 Temperature Probe (Campbell Scientific, Inc., Logan, UT, USA) in two depths: 0.05 and 0.10 m. Rainfall was measured by a TB4 rain gauge (Campbell Scientific, Inc., Logan, UT, USA). All sensors were installed at a 11 m height above the soil surface, except for the ones below the ground. These data were sampled every 5 s and stored as half-hourly means.

### Data processing

#### Net ecosystem exchange

The NEE is the sum of the CO_2_ turbulent flux (${{\rm{F}}}_{{{\rm{CO}}}_{2}}$), measured through the covariance between fluctuations in the vertical wind velocity (w^′^) and CO_2_ density (c^′^), and the change of CO_2_ storage in the air column below the EC measuring height (${\rm{Sc}}$), i.e.:

1 $${\rm{NEE}}={{\rm{F}}}_{{{\rm{CO}}}_{2}}+{\rm{Sc}}\to ({{\rm{\mu }}{\rm{molm}}}^{-2}{{\rm{s}}}^{-1})$$

where ${F}_{{{\rm{CO}}}_{2}}\,$was calculated through the following equation described in the study^69^:

2 $${{\rm{F}}}_{{{\rm{CO}}}_{2}}={\rho }_{{\rm{air}}}\cdot \overline{w{\prime} c{\prime} }\to ({{\rm{\mu }}{\rm{molm}}}^{-2}{{\rm{s}}}^{-1})$$

where ${\rho }_{{\rm{air}}}$ is the air density and $\overline{w{\prime} c{\prime} }$ the covariance between fluctuations in the vertical wind velocity and CO_2_ density.

Half-hourly means of ${\rm{Sc}}$ were calculated using the method proposed in the literature^70^ and vastly used in subsequent studies^8,71,72^. Because no concentration profile was installed at the site, we opted for the discrete approach, which considers CO_2_ concentration inside the canopy as constant, which in turn represents only an approximation^72^:

3 $$\begin{array}{ccc}{\rm{Sc}}=\frac{\Delta {{\rm{C}}}_{{{\rm{CO}}}_{2}\cdot }{\rm{z}}}{({\rm{R}}\cdot {{\rm{T}}}_{{\rm{a}}}/{{\rm{P}}}_{{\rm{a}}})\cdot \Delta {\rm{t}}} & \to  & ({{\rm{\mu }}{\rm{molm}}}^{-2}{{\rm{s}}}^{-1})\end{array}$$

where $\Delta {C}_{C{O}_{2}\cdot }\,$is the change in CO_2_ concentration $({{\rm{\mu }}{\rm{molm}}}^{-2}{{\rm{s}}}^{-1})$, $z$ is the height of the EC system above the ground (m), $R$ is the universal gas constant, T_a_ is air temperature (K), is ambient air pressure over the 30-min interval $\varDelta t$ (s).

The ${{\rm{F}}}_{{{\rm{CO}}}_{2}}$ values were calculated using the LoggerNet software (Campbell Scientific, Inc., Logan, UT, USA) by converting the high frequency data into the binary format (TOB1) with a 30 minutes timestep. Afterwards, data were processed using the EdiRe software (http://www.geos.ed.ac.uk/abs/research/micromet/EdiRe/). The EdiRe algorithm transforms high frequency data in half-hourly means, also including a series of corrections: detection of spikes, delay correction of H_2_O/CO_2_ in relation to the vertical wind component, coordinates rotation (2D rotation) using the planar fit method, sonic virtual temperature correction, corrections for density fluctuation (WPL correction) and frequency response correction.

### Data quality control and outlier detection

Data post-processing was conducted in three steps: (i) data quality assessment, by rejecting low quality data, data associated with sensor mal-functioning and visibly inconsistent data; (ii) data were submitted to a robust outlier detection algorithm, as proposed in the literature^73^; (iii) due to low turbulence conditions during the night, all nighttime flux data were rejected if friction velocity (u_*_) was below a critical threshold (from 0.18 to 0.34 m s^-1^)^21^. The u_*_ threshold was determined based on the moving point test (MPT) applied on nighttime data^73^. In order to eliminate spurious fluctuations on CO_2_ and energy fluxes data we used an algorithm based on moving medians for the identification of spikes. This method consists of separating the data series into a smooth part and a residual part, and manually removing all spurious data. Data gaps originated by removing spikes were filled using a marginal distribution sampling (MDS) algorithm which considers not only the covariation between fluxes and meteorological data but also temporal auto-correlation of fluxes^74^. In this algorithm, actions are taken considering the following conditions: i) if there are missing flux data, but meteorological data (incoming solar radiation – R_g_, T_a_ and vapor pressure deficit – VPD) are available, then the gap is filled with the mean value considering similar meteorological conditions in a 7-day window; ii) if only incoming solar radiation data are available, the gap is filled with the mean value considering similar meteorological conditions in a 7-day window; iii) if no meteorological data are available, the gap is filled by the mean value in the last hour, and thus considering diurnal variation of each variable. If data gaps still exist after applying the algorithm, the same procedures will be carried out but considering larger time windows. The gap filling method was carried out by using an online tool by the Max Planck Institute (Max Planck Institute for Biogeochemistry - http://www.bgc-jena.mpg.de/~MDIwork/eddyproc/).

### Carbon balance

Carbon dioxide fluxes were partitioned in order to separate NEE into GPP and R_eco_. We used a flux partitioning method as described in the literature^74^. For nighttime periods, we considered GPP to be zero and therefore NEE was estimated as follows:

4 $${\rm{NEE}}={{\rm{R}}}_{{\rm{eco}}},\,{\rm{for}}\,{\rm{nighttime}}\,{\rm{periods}}$$

5 $${\rm{NEE}}={{\rm{R}}}_{{\rm{eco}}}-{\rm{GPP}},\,{\rm{for}}\,{\rm{daytime}}\,{\rm{periods}}$$

Nighttime fluxes were adjusted in relation to T_a_ through the equation^75^:

6 $${{\rm{R}}}_{{\rm{eco}}}={{\rm{R}}}_{{\rm{eco}}{\rm{.ref}}}\cdot exp\left({E}_{0}\cdot \left(\frac{1}{{T}_{ref}-{T}_{0}}-\frac{1}{{T}_{air}-{T}_{0}}\right)\right)$$

where R_eco_ (μmol m^−2^ s^−1^) is the sum of autotrophic and heterotrophic respiration rates, R_eco.ref_ is the respiration rate at a reference temperature T_ref_ (15 °C), E_0_ (K) is the activation energy or the R_eco_ dependency on temperature expressed as a temperature value, and T_0_ is the baseline temperature adjusted to −42.02 °C. This model relates R_eco_ to T_a_ for nighttime data and the obtained function is then used to extrapolate R_eco_ values for daytime periods. Both R_eco_ and GPP were calculated using the online tool by the Max Planck Institute (Max Planck Institute for Biogeochemistry - http://www.bgc-jena.mpg.de/~MDIwork/eddyproc/). It is worth mentioning that we did not incorporate to the CO_2_ flux partitioning procedure any method to detect apparent ecosystem-scale inhibition of daytime respiration, which could overestimate GPP as suggested in recent findings^76^.

The light response of NEE was evaluated. The NEE daytime data based estimate was modeled using the common rectangular hyperbolic light–response curve^60^:

7 $$NEE=\frac{\alpha \cdot \beta \cdot {R}_{g}}{\alpha \cdot {R}_{g}+\beta }+\gamma $$

where ${\rm{\alpha }}\,({\rm{\mu }}{\rm{mol}}\,{{\rm{CJ}}}^{-1})$ is the light use efficiency and represents the initial slope of the light response curve, ${\rm{\beta }}\,({\rm{\mu }}{\rm{mol}}\,{\rm{C}}\,{{\rm{m}}}^{-2}{{\rm{s}}}^{-1})$ is the maximum CO_2_ absorption rate of the canopy at light saturation, ${\rm{\gamma }}\,({\rm{\mu }}{\rm{mol}}\,{\rm{C}}\,{{\rm{m}}}^{-2}{{\rm{s}}}^{-1})$ is the ecosystem respiration and ${{\rm{R}}}_{{\rm{g}}}\,(W{m}^{-2})$ is incoming solar radiation.

The mean ${{\rm{NEE}}}_{{\rm{Midday}}}$ was calculated between 10:00 and 12:00 (local time), while mean ${{\rm{NEE}}}_{{\rm{Night}}}$ was calculated between 20:00 and 22:00 (local time). During these periods data were stable, presenting little to no variability.

### Footprint calculation

The flux footprint was calculated using the two-dimensional parameterization model called Flux Footprint Prediction^77^. This model requires the following data: flux measurement height (zm = 11 m), zero-plan displacement (d), surface friction velocity (u*), vertical wind velocity deviation (σw), and roughness length (z0m). In our study site canopy hight (h) was of 6 m. However, this parametrization is valid for moderate friction velocity values (u*> 0.1 m s-1) and for a limited range of boundary layer stability conditions (−15.5 ≤ zm/L) where L is Monin-Obukhov length^77^. In our study area, previous studies^21^ considered d = (2/3).h and z0 = 0.123.h.

### Vegetation state

In order to evaluate the seasonality of vegetation cover in response to the seasonal variability of rainfall, we used the Enhanced Vegetation Index (EVI) obtained through the MOD13Q1 product from the Moderate Resolution Imaging Spectroradiometer (MODIS), onboard the Terra satellite (United States Geological Survey) (https://earthexplorer.usgs.gov). EVI data have been frequently used to assess the effects of vegetation conditions on the closure of the energy balance and CO_2_ exchanges^78^.

### Statistical analysis

The daily means and totals of the meteorological variables and CO_2_ flux components were bootstrapped over seasonal intervals for the estimation of random variance (± 95% of confidence interval – CI) about the mean according to the methodology presented in the literature^24^. Statistically significant differences (p < 0.05) in the mean seasonal value for a given meteorological variable or CO_2_ flux components were determined by the degree of overlap in the 95% bootstrapped CI^26^. The correlation matrix heatmap (Pearson’s correlation test) was used for examining the relationships among meteorological variables and CO_2_ flux components. Additionally, the model coefficients were tested under the null hypothesis at a 5% significance level. All statistical analysis was carried out using the R software^79^.
